# A typology of caregiving spouses of geriatric patients without dementia: caring, worried, desperate

**DOI:** 10.1186/s12877-021-02425-1

**Published:** 2021-09-06

**Authors:** Thomas Johann Gehr, Ellen Freiberger, Cornel Christian Sieber, Sabine Alexandra Engel

**Affiliations:** 1 Institute for Biomedicine of Aging, Friedrich-Alexander-University Erlangen-Nürnberg, Kobergerstraße 60, 90408 Nürnberg, Germany; 2 Hospital of the Order of St. John of God Regensburg, Regensburg, Germany; 3 Department of Internal Medicine, Kantonsspital Winterthur, Winterthur, Switzerland; 4 Department of Social Services, Catholic University of Applied Sciences of North Rhine-Westphalia, Campus Paderborn, Paderborn, Germany; 5 Private Institute for Gerontological Intervention and EduKation at Dementia GmbH, Buckenhof, Germany

**Keywords:** Typology, Caregiver, Partner, Burden, Coping

## Abstract

**Background:**

An increasing number of older people in Germany receive care at home from family members, particularly from spouses. Family care has been associated not only with subjective burden but also with negative effects on caregivers’ health. A heterogeneous group, caregivers are confronted with individual situational demands and use different available coping strategies. To date, little is known about the relationship between burden and coping by spousal caregivers, particularly in the context of geriatric patients without dementia.

**Objectives:**

The aim of this study is to explore the burden and coping strategies of caregiving spouses of geriatric patients without dementia and with a hospitalization within the last year. To help explore this population, a typology is presented that has been based on reported perceptions of home care burden and individual coping strategies. Furthermore, a case study is presented for each type of spousal caregiver.

**Methods:**

The study used a concurrent mixed method design with a sample of nine spousal caregivers (mean age: 78.9 years). Four women and five men were recruited in an acute hospital setting during the TIGER study. Quantitative data were collected using a self-questionnaire and qualitative data were gathered through nine problem-centered interviews with spousal caregivers. The latter were subsequently analyzed utilizing the structured content analysis method. The data were then summarized to nine individual cases. Finally, the results were clustered using the empirically grounded construction of types and typologies. Each type of spousal caregiver is presented by a case study.

**Results:**

Three types of caregiving spouses were identified: “The Caring Partner”, “The Worried Manager” and “The Desperate Overburdened”. These types differ primarily in the level of subjective burden and caregiving stress, the coping strategies, the motivation for caregiving, and expressed emotions.

**Conclusions:**

The development of this new typology of caregiving spouses could help health care professionals better understand caregiving arrangements and thus provide more targeted advice.

**Trial registration:**

The TIGER study was registered with clinicaltrials.gov: NCT03513159. Registered on April 17, 2018.

**Supplementary Information:**

The online version contains supplementary material available at 10.1186/s12877-021-02425-1.

## Background

Due to Germany’s aging population, the role of informal caregivers is becoming increasingly important in providing necessary support for older patients. Of the more than 4.25 million care recipients in Germany who were legally dependent on long-term care, 79% were living at home in 2019. More than 90% of this population received help and care from informal caregivers, in most cases a family member [1]. Family carers are the main source of support for older patients living at home, making them Germany’s largest “nursing service” [2]. Most of those providing care for an older person are spouses/partners (48%) with an average age of 75.8 years [3]. The term spouse in this paper thus encompasses husbands and wives as well as unmarried partners living together in a long-term partnership.

Caregivers have been described as “hidden patients” [4]. Compared to non-caregivers, the caregiver burden has not only been associated with worse health outcomes (e.g. depression, anxiety, stress, physical health), but has also been associated with decreased feelings of self-efficacy and poorer general subjective well-being [5–7]. Zarit et al. defined the caregiver burden “as the extent to which caregivers perceived their emotional or physical health, social life, and financial status as suffering as a result of caring for their relative” [8]. Various studies have examined the burden of caregivers in terms of disease, age, gender, relationship, housing, work, social support and re-hospitalization [5, 7, 9–11]. Thus, caregiver burden can be seen as a multidimensional construct ranging from objective burdens and self-reported stress burdens to relationship burdens [12].

Despite these negative findings, caregivers also reported positive aspects to caregiving [13], so-called uplifts [14]. Reported benefits of caregiving have further included feeling needed; acquiring new skills; a perceived increase in personal importance; being able to take responsibility; and the joy of spending time with the feeling to be needed [15]. This suggests that the care of a relative must not automatically be considered a negative experience.

Studies examined the difference between caregiving spouses and caregiving children. They reported that spouses seem to generally provide more care but use less professional support, perceive their physical health to be worse, experience more burden and depression, and have a lower sense of wellbeing and self-efficacy [5, 7, 16].

Regarding the group of caregiving spouses with positive caregiving experiences, caregiving was described as a responsibility and a source of personal growth, a gift of fulfilling commitment to one another [17]. Those with negative experiences reported a perceived lack of choice in the matter of caregiving [18]. Perceived caregiver burden thus depends, at least partially, on the coping strategies of the individual caregiver.

The transactional theory of stress and coping by Lazarus and Folkmann [19] has often been used as a theoretical framework for understanding caregiver distress. The authors defined coping as “constantly changing cognitive and behavioural efforts to manage specific external and/or internal demands that are appraised as taxing or exceeding the resources of the person” [19]. This process includes various different coping strategies. Heim et al. developed the Bernese Coping Modes (BEFO), a measurement instrument based on the transactional theory of stress and coping, as well as clinical observations [20, 21]. The BEFO divides disease-oriented coping into three general dimensions: cognitive, emotional, and behavioural strategies. This instrument has proved to be applicable across a wide range of diagnostic areas or contexts [20], including the context of family caregiving [22]. A factor analysis of the BEFO identified three coping dimensions: ‘diverting’, ‘negative emotional’ and ‘seeking attention and care’ [23]. According to Gunzelmann et al., who investigated coping dimensions in the context disease of older persons, good subjective health and emotional support are associated with ‘diverting’; a high level of social burden and practical social support are associated with ‘seeking attention and care’; and ‘negative emotional’ coping can be accordingly found with high social burden and poor subjective health [24]. To date, these coping dimensions by Hessel et al. [23] have not been applied to the context of family caregivers, specifically spouses.

Many studies on the coping strategies of caregiving relatives have not considered the heterogeneity of this group. Caregivers are confronted with individual situational demands and use different available coping strategies. Therefore, caregivers should be considered as an aggregation of several different subtypes of caregivers that differ in the coping strategies they choose [25]. Even less is known about the relationship between burden and coping by spousal caregivers [17], especially for those of geriatric patients without dementia. Previous typological studies with caring spouses described the experiences across different groups of neurodegenerative diseases, particularly dementia and Parkinson disease [26–29].

To our knowledge, no typology exists for caregiving spouses of geriatric patients without dementia. A typology for this special group could support health care professionals in better understanding individual needs and providing more targeted advice.

### Aim

The aim of this study was to explore the burden and coping strategies of caregiving spouses of geriatric patients without dementia and a hospitalization within the last year. To help explore this population, a typology is presented that has been based on reported perceptions of home care burden and individual coping strategies. Furthermore, a case study is presented for each type of spousal caregiver.

## Methods

### Design

A concurrent mixed method design, involving two identical samples for both quantitative and qualitative components of the investigation, was used. The single-case data were subsequently aggregated to search for patterns that extended beyond a single case and to derive more general findings. To reduce the heterogeneity and generalize the empirical data for individual cases, results were clustered using the empirically-grounded construction of types and typologies [30, 31]. The combination of quantitative instruments for measuring psychosocial factors and qualitative interviews in this study allowed more in-depth exploration of how spousal caregivers cope with the everyday life care of their partner.

### Sample

Caregiving relatives were recruited from selected participants in the *Transsectoral Intervention Program for Improvement of Geriatric Care in Regensburg Study* (TIGER) (ClinicalTrials.gov ID: NCT03513159). The study protocol was approved by the ethic committee of the Friedrich-Alexander-Universität Erlangen-Nürnberg, Germany. The aim of the TIGER Study was to reduce hospital readmission rate among geriatric patients. Participants were thereby individually supported by specialized geriatric nurses for up to 1 year following hospitalization. Patient inclusion criteria were a minimum age of 75 years, a Mini Mental State Score of more than 22. Exclusion criteria were a palliative status, and a planned hospital readmission within the next 4 weeks [32].

Relatives of the TIGER participants were approached in the hospital or within a few days after to obtain written informed consent to participate as primary caregivers. A total of 59 relatives participated in the TIGER Study. For the present study, we selected those who met the following inclusion criteria: (a) being a spouse or partner in a long-term relationship and sharing a common household (b) being the main caregiver and (c) the care recipients had been classified as a minimum degree two regarding long-term care need (‘Pflegegrad’). The degree of long-term care need (‘Pflegegrad’) in Germany is set according to criteria listed in the German Social Code XI [33], with the individual degree appraised by the health insurance medical service (MDK). There are five degrees, with higher degrees indicating a higher care dependency. Degree two signifies a significant impairment to independent living.

**Fig. 1:**
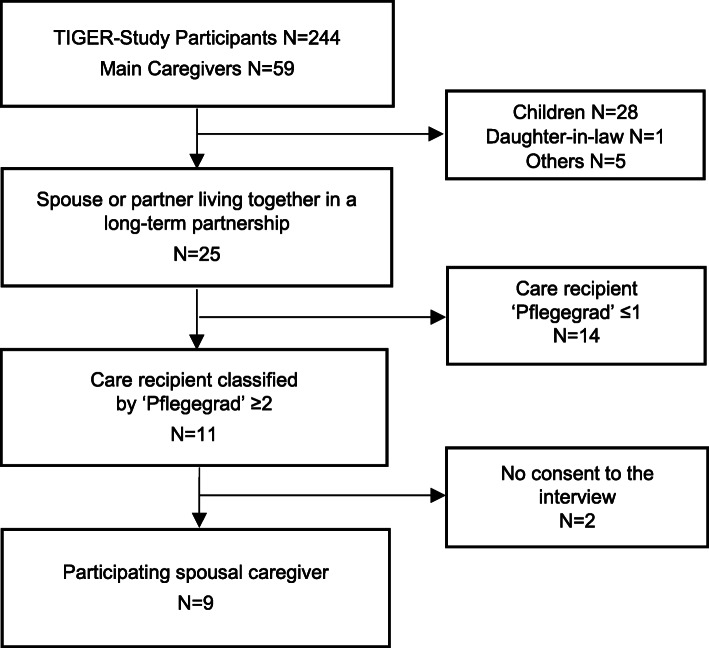
Flow chart recruited caregivers

For the current study, nine spousal caregivers were recruited (see Fig. 1).

### Data collection and data analysis

**Fig. 2:**
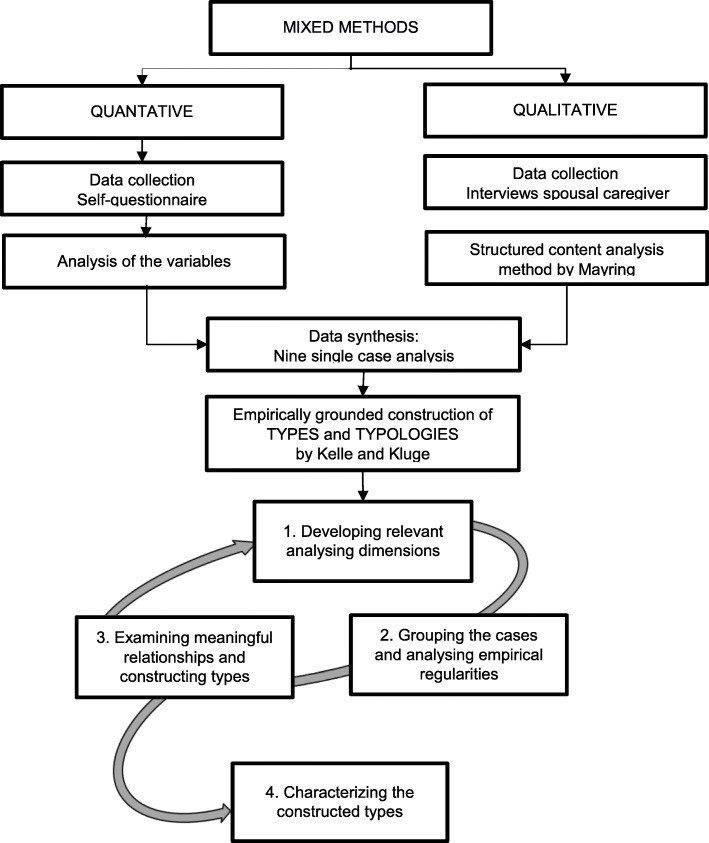
Description of the exploratory design

Figure 2 illustrates the evaluation process from the collection of quantitative and qualitative data, to single case analysis, and finally the creation of a caregiver typology.

#### Quantitative data

Caregiver data were collected using demographic information and a validated self-questionnaire from the TIGER Study. The current analysis focused on the following data:

- **Demographics***:* Age, sex, education level, social situation, degree of dependency on long-term care (‘Pflegegrad’).
- **Caregiver burden:** Caregiver burden was measured using the *Zarit Burden Interview* (ZBI), which consists of 22 self-report items. Total scores range from 0 to 88, with higher scores indicating a higher perceived burden [8]. A score of 0–20 can be categorized as little or no burden, 21–40 as mild to moderate burden, 41–60 as moderate to severe burden, and 61–88 as severe burden.
- **Experienced stress***:* The German Version of the *Perceived Stress Questionnaire* (PSQ) is a validated instrument with 20 items used to assess subjectively experienced stress [34]. The resulting score can range from 0, the lowest level of perceived stress, to the highest level of perceived stress: 1.
- **Expressed emotions** (EE)*:* The *Family Questionnaire* (FQ) assessed two scores: criticism (CC) and emotional over-involvement (EOI)*.* The caregiver attitude towards his or her partner can be rated as very critical or emotionally over-involved if the sum score of the scale exceeds the given cut-off points [35].
- **Health-related quality of life:** The *12-Item Short Form Health Survey* (SF-12), with a mental (MCS) and physical component summary (PCS) score, was used to evaluate health-related quality of life. Scores and can range from 0 to 100. Higher scores indicate better health status [36].

Descriptive statistics regarding quantitative data were obtained using the Statistical Package for Social Sciences (IBM SPSS V26).

#### Qualitative data

From October 2019 to February 2020, the first author conducted nine problem-centred interviews [37] with spousal caregivers. Interviews took place apart from the patient and in the home of the spousal caregiver, between one and ten months following hospital discharge. The developed semi-structured interview guide (Additional file 1) covered the demands of care, caregiver motivation, the perceived burden and uplifts of caregiving, and the personal coping style of the caregiving spouse. Additional prompts were used when appropriate to elicit more information about each topic. The interview guide was not rigidly adhered to, leaving room for an open and reflective dialogue. The interviews lasted 42–112 min, were audio-recorded, transcribed verbatim and pseudonymized [38]. The transcripts were transferred to the qualitative data analysis software MAXQDA (VERBI Software, Berlin, Germany). Each transcript was read soon after the interview to gain a sense for the whole. The interviews were analysed following the structured content analysis method by Mayring [39]. A deductive-inductive approach was used to discover the main response categories listed above. The coping style was further analysed on the basis of BEFO and broken down into 28 subcategories of coping-modes, which could be classified under the dimensions ‘diverting’, ‘negative emotional’ and ‘seeking attention and care’ [23].

Both quantitative and qualitative data were summarized in a matrix to obtain an overview of the nine individual cases. This served as the basis for the next step of analysis: the constructing a typology.

#### Construction of a typology

A typology is defined as “the result of a grouping process: An object field is divided in some groups or types with the help of one or more attributes” [31]. Kelle and Kluge have described four analytical stages to the construction of a typology (Fig. 2) [30]. Based on the theoretical background and collected data, this current study thus chose the ‘subjective caregiver burden’ and reported ‘caregiver coping behaviour’ as relevant attributes in identifying similarities and differences between the nine single cases (Stage 1). The nine individual cases were then clustered into groups according to the pre-defined attributes of the ZBI and the dimensions of the BEFO. For this reason, the three dimensions according to Hessel et al. [23] were ranked according to the predominant form for each case.

Using an Excel database, individual caregiver burdens, coping styles, and further attributes were cross-referenced to facilitate within-case and between-case analyses (Stage 2). To obtain a deeper understanding of the social aspect, the relationships between the selected attributes and the constructed types were then explored (Stage 3). ‘Caregiver motivation’ and ‘expressed emotions’ were further included to augment understanding of differences between the groups and find similarities within the groups. As illustrated in Fig. 2, these three stages occurred in a constant feedback loop. As a last step, three cases, best exemplifying the selected attributes of burden and coping style, were selected to illustrate the three constructed types presented in this paper (Stage 4). Ongoing discussion with experts of alternative ways of interpretation throughout the research process was used to increase the validity of the findings.

## Results

### Description of the sample

The final sample (Table 1) consisted of nine spousal caregivers, four women and five men, with a mean age of 78.9 years. All couples lived in an apartment or house without any other supporting family members in the close vicinity. The care recipients had a degree of care dependency (‘Pflegegrad’) between 2 and 5 and a MMST between 25 and 30 (Mean MMST: 27.7). In the interviews, the spouses reported at least two hospital stays of their partner during the last year. Four couples received regular care support through an outpatient care service, three received support through relatives, and two received support from both relatives and an outpatient care service.

**Table 1:** Participant characteristics

Name Age Range	Care recipient		Caregiving Motivation	ZBI	EE		COPING Dimensions	PSQ	SF-12	
	Gender Age Range	‘Pflege-grad’			EOI	CC		Total	PCS	MCS
Mr. A. 80–84	F 75–79	2	affection	no or little burden	low	low	1. Diverting	.12	55	60
Mr. B 80–84	F 85–90	2	affection	mild to moderate burden	low	low	1. Diverting 2. Seeking attention & care 3. Negative emotional	.20	52	40
Mrs. C 75–79	M 85–90	5	affection	mild to moderate burden	low	low	1. Diverting 2. Seeking attention & care 3. negative emotional	.43	34	41
Mrs. D 75–79	M 80–84	3	obligation	mild to moderate burden	high	high	1. Seeking attention & care 2. diverting	.45	37	46
Mrs. E 80–84	M 90–94	3	affection	mild to moderate burden	high	low	1. Seeking attention & care 2. Negative emotional 3. Diverting	.60	47	53
Mr. F 80–84	F 80–84	4	affection	moderate to severe burden	high	low	1. Seeking attention & care 2. Diverting 3. Negative emotional	.62	40	31
Mr. G 85–90	F 75–79	5	obligation	moderate to severe burden	high	low	1. Negative emotional 2. Diverting 3. Seeking attention & care	.70	44	45
Mr. H 75–79	F 75–79	2	obligation	moderate to severe burden	high	high	1. Negative emotional 2. Seeking attention & care 3. Diverting	.70	29	29
Mrs. J 60–65	M 75–79	2	obligation	moderate to severe burden	high	low	1. Negative emotional 2. Seeking attention & care	.92	37	19

Pflegegrad = Degree of dependency on long-term care; *ZBI* Zarit Burden Interview, *EE* Expressed emotions, *EOI* Emotional over-involvement, *CC* Criticism, *PSQ* Perceived Stress Questionnaire, *SF-12* 12-Item Short-Form Health Survey, *PCS* Physical component score, *MCS* Mental component score, *F* female, *M* Male;

Five spouses described affection as their motivation for taking over care, and four spouses reported obligation as their motivation. Following the ZBI classifications, one participant ranked the care burden as no or little burden, and four participants each ranked the care burden as either mild to moderate or moderate to severe. Two-thirds of the FQ respondents had a high emotional over-involvement (EOI); two participants additionally had a high criticism (CC) towards her partner. The coping strategies for most of the spouses could be attributed to all three dimensions of the BEFO, one participant from each of one or two dimensions. The subjectively experienced stress measured by the PSQ showed a broad range (Score = .12–.92; Mean = .53). The self-reported health-related quality of life measured by the SF-12 was 42 (range: 29–55) for the physical dimension and 40 (range: 19–60) for the mental dimension.

### Typology of caregiving spouses

By defining three types of spousal caregiver, an association between the caregiver burden and the applied coping strategies could be identified. Correlating attributes to contrast the types were the ‘motivation to care’ and the ‘expressed emotions’. Other checked attributes such as ‘gender’, ‘duration of care’, ‘degree of care’ or ‘type of support’ were checked had no influence on the development of the typology. Furthermore, two participants receiving high CC and high EOI scores had expressed the suspicion that their spouses had dementia. The PSQ supported our presented typology by increasing stress levels from Type A to Type C.

Type A demonstrated little to moderate burden, the lowest stress level of the three types and low expressed emotions. The predominant coping dimension was ‘diverting’. Type A can be characterized by the motivation to care due to feelings of affection. Mr. A, Mr. B and Mrs. C were assigned to this type.

Type B demonstrated mild to severe burden, a higher stress level than Type A and emotional over-involvement. The predominant coping dimension was ‘seeking attention and care’. The motivation to care for this Type differs. Mrs. D, Mrs. E and Mr. F were assigned to this type.

Type C demonstrated moderate to severe burden, the highest stress level and high emotional over-involvement. The predominant coping dimension was ‘negative emotional’. Type C can be characterized by the motivation to care due to feelings of obligation. Mr. G, Mr. H and Mrs. J were assigned to this type.

Burdened caring partners additionally tended to report more health impairments and showed a lower health-related quality of life.

**Fig. 3:**
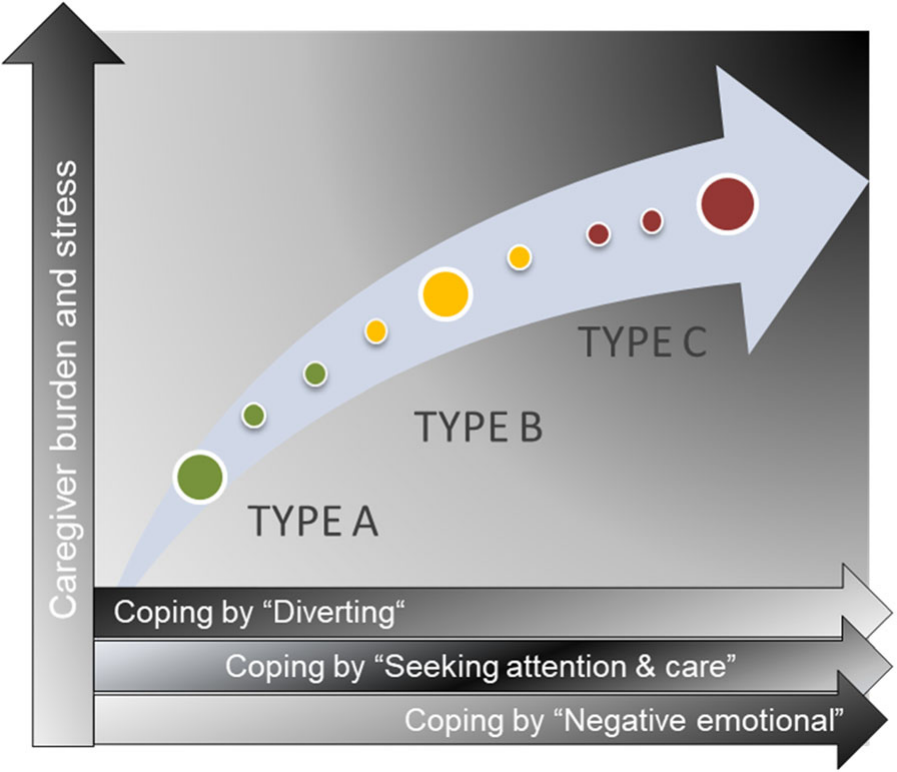
Typology of caregiving spouses by caregiver burden and the coping dimension. The vertical axis shows the extent of experienced burden and stress. A curved arrow-symbol in the graphic growing bigger upwards indicates the level of burden too. In the horizontal axis the respective coping dimensions are plotted. The big green dot represents the prototypical case of coping-type ‘Diverting’, the big yellow dot the prototypical case of coping-type ‘Seeking attention and care’ and the big red dot the prototypical case of coping-type ‘Negative emotional’. One always has to keep in mind also in the prototypes of our participants parts of the other two coping dimensions might also be present, but to a much lesser extent. The smaller dots symbolize mixed forms of coping strategies, whereby the colour indicates the predominant coping dimension

Figure 3 illustrates the correlation between perceived burden and stress and the three dimensions of coping.

For the following description, we selected one prototypical representative per each spousal caregiver type [30] to illustrate our findings.

#### Type A: “The Caring Partner”

Mr. A cares for his wife. They had been married for about 60 years and were self-employed together. They had little contact with their relatives. The couple lived in a barrier-free rented flat. For the last 2 years, Mrs. A has been dependent on increased support and care, with the last hospital stay 9 months ago due to chronic lung disease. When his wife “*was not there, it was gloomy. Then I look where she always sits or lies, […], she is not here”* (Mr. A, para. 84). She used a wheeled walker because of a gait and balance disorder. She was also urinary incontinence and impaired vision and hearing. In the morning, she was cared for by an ambulant nursing service. Her husband provides the following: Assistance walking, going to the toilet, dressing, household, accompaniment to the doctor, preparation of medication, and insulin application.

The war and the post-war years were formative for Mr. A: *“It’s from all the experiences of the war, when your father was at war and your mother was alone at home, and when you somehow notice something like that, you think in a different way and then you see that everything turned out well. Then you say, do not give up courage, not hope”* (Mr. A, para. 94). Mr. A relativized his own situation by comparing it with the past. His father had also cared his wife: *“But he never complained. He never complained. How many more years did he care for her? 5-6 years at least. Yes, yes. Mother needed a lot of care” (Mr. A, para. 98). Mr. A considered supporting his wife “a matter of course and we have said yes, in good times and bad times”* (Mr. A, para. 38). Moreover, his wife supported him by giving him instructions for household activities: *“Everything works out quite well. We are already a well-coordinated team”* (Mr. A, para. 19).

During the interview, Mr. A repeatedly emphasised how important being together is to him. Humour was important for living together: *“We laugh a lot”* (Mr. A, para. 140). He was also pleased with the respect and appreciation he receives from his wife: *“And she is really happy that she has me. And I am glad that I have her. She then says: You did a good job or it’s good that I have you and all that”* (Mr. A, para. 74). Mr. A tried to suppress thoughts about the future and death with positive thoughts: *“You should rather have beautiful thoughts. I prefer to be optimistic. That makes you not want to look so far ahead. That comes by itself”* (Mr. A, para. 46).

In Mr. A’s view, partnership and cohesion were of central importance. This had been a feature of his life since childhood. He saw caring for his wife not as a burden but as a matter of course. He could not describe any stressful situations in everyday life. The times when he was separated from his wife were most likely to be burdensome for him. He tried to avoid these situations by taking care of his wife and he tried to suppress thoughts of death. He wished *“that things stay the way they are and that we both stick together”* (Mr. A, para. 162). The expressed coping styles ‘acceptance’, ‘preserving composure’, ‘relativizing’, ‘self-validation’, ‘humour’, ‘optimism’ and ‘suppression’ by Mr. A were related primarily to the ‘diverting’ coping dimension.

#### Type B: “The Worried Manager”

Couple E had already been married for about 50 years and had little contact with their only child. They lived in a rented, non-barrier-free apartment. Mr. E had been hospitalized twice in the last year for falls, in which he suffered painful injuries. He also suffered from severe itching, gait disorder and incontinence, as well as impaired vision and hearing. Mrs. E supported her husband with personal hygiene, dressing, walking with the wheeled walker and medication. She had also taken over all organizational tasks and the household. The extent of the support was also dependent on Mr. E’s daily form. Once a week, Mrs. E received support from a housekeeping as well as an outpatient nursing service to help her husband shower.

The organization and bureaucracy of the healthcare situation was particularly stressful for Mrs. E. This is also due to the fact that without a car, trips to public agencies or doctors were a great challenge for the couple: *“Visits to doctors cost, you could say, half a day. Because after that he is done. And me too sometimes”* (Mrs. E, para. 29). Recently she purchased a medical alert system: *“I always have my cell phone with me, but when I know he has the medical alert button I just feel safer”* (Mrs. E, para. 25). She mentioned that it was not easy for her to ask others for support: *“But I don’t like to make myself so dependent. Which is perhaps to my own detriment, I honestly admit”* (Mrs. E, para. 17).

The housekeeping service was rather a stress factor for her: *“I’ve always been glad when that’s over on Monday”* (Mrs. E, para. 53). There were also situations where she avoided being active. Regarding her husband’s increasing visual impairment she said: *“It is not that I am not dealing with the situation, […], I don’t know how to explain it”* (Mrs. E, para. 95).

A lack of understanding for Mrs. E’s situation involved *“frustration. Stress. Stress”* (Mrs. E, para. 37). *“Today it is so and tomorrow it can be […]. And do you know what the worst thing is? Nobody believes you that the differences are so great”* (Mrs. E, para. 33). Mrs. E had reduced social contacts to those that were positive for her: *“And just gossiping over the garden fence and maligning people is a waste of my time”* (Mrs. E, para. 45).

She often felt very sorry for herself, especially because of the limited free time due to reduced mobility. This caused *“that I get an outburst of rage. That I am currently beginning to scold. Well, that can happen to me, I’m quite honest”* (Mrs. E, para. 59). *“Such a situation comes and then it is over again”* (Mrs. E, para. 63). In addition, she also tried to accept her situation by relativizing: *“And there are certainly those who are worse off, I am quite sure of it*” (Mrs. E, para. 95). Another active way of dealing with stress was *“chasing the problems away by walking. You then see things a little differently”* (Mrs. E, para. 71). She was able to divert and relax by *“beautiful music, nice things on TV. And often also a little walk. Maybe inconspicuous for everyone else, but for us it is something”* (Mrs. E, para. 67).

She described the relationship with her husband as a source of strength and purpose: *“And because we are there for each other. It has been like that, recently, when I was not feeling very well and my husband was there, he supported me. If we have luck, how to say, if God wills, we will have a golden wedding next year. […] and that we are there for each other. That’s why we got married”* (Mrs. E, para. 39).

Mrs. E also found consolation and reassurance *“when I can go to church*” (Mrs. E, para. 45). She appreciated the conversation with the priest: *“We can laugh together […] and on the other hand we can be really serious with each other”* (Mrs. E, para.47).

In Mrs. E’s case, all three coping dimensions could be seen. However, the coping dimension ‘seeking attention and care’ was the most pronounced in her descriptions. These included ‘emotional release’, ‘attention and care’, ‘giving meaning’, and ‘religion’. ‘Social withdrawal’ as a focus on oneself was also part of it. ‘Negative emotional coping styles in the form of ‘active avoidance’, ‘self-pity’ or ‘release of anger’ served primarily to release her from the demands on herself. ‘Relativizing’ and ‘valorising’ her situation were used as coping dimension ‘diverting’.

#### Type C: “The Desperate Overburdened”

Mr. and Mrs. J had been married for about 40 years and lived in their own house. The couple had been self-employed. Their only child currently lives further away but visits her parents almost weekly to support them. In addition to various internal diseases, Mr. J had a stroke some years ago. He had a high risk of falling and therefore used a wheeled walker. His last hospitalization was for clarification of syncope.

Mrs. J felt strongly burdened by the care and support of her husband. She repeatedly described the permanent availability and the fear of falling as extremely stressful. *“You listen, you hear all this, you hear the toilet flush, you think, hopefully it works. Then you hear the banging at the door again, because that’s what happens. Actually a permanent fear”* (Mrs. J, para. 43). “Always the permanent listening, at night in standby” (Mrs. J, para. 29). She had quite different expectations of the time spent together in later life, *“because I didn’t imagine that we would be hanging on each other all the time”* (Mrs. J, para. 37).

Friends of her husband would take him along to events or excursions from time to time. *“Then I am incredibly grateful and very, very happy and I have an evening for myself”* (Mrs. J, para. 71).

The last hospital stays of her husband had been *“terrible” in her eyes. “Well, I could sleep pretty well at night, because I knew he was safe. But in the daytime, what should I expect when I go there? What do I have to take with me? What do I have to think about? Laundry brought in, other laundry taken out. The driving in, the parking, […]. So there were three hours away like nothing. And that was again the loss of time. You sit there at half past seven in the evening […] actually you are exhausted and tired”* (Mrs. J, para. 65). The lack of time was a key issue for Mrs. J.

Mrs. J described everyday life as *“dragging along, letting such a time pass, such waiting, now I do this quickly, but then he is tired”* (Mrs. J, para. 13), *“at some point you become speechless, because what do you still want to talk about?”* (Mrs. J, para. 65). She also appeared to be resigned. She was no longer interested in activities like going to the cinema or meeting her friends. In her circle of acquaintances she felt little recognition and understanding: *“Actually, only those who have similar situations at home or are just as restricted can understand this”* (Mrs. J, para. 182).

Mrs. J had the feeling that she had to do everything on her own: *“My husband always says we have to do it. So then I also know what he wants, but in the end it’s just me. We have to do it”* (Mrs. J, para. 108). Overall, she criticized that her husband showed too little engagement in performing therapeutic exercises.

With her child, Mrs. J can talk about her feelings: *“It is actually a sadness.* [Name of the child] *sometimes says, you don’t get angry then? And then I said, no, I don’t feel aggression and anger yet. Just, oh my God, this sadness, but I am also taking antidepressants”* (Mrs. J, para. 27).

Nevertheless, Mrs. J tried to find some time for herself at home, which was difficult for her*: “Talk to myself and try to distract myself, including room scents, aromatherapy, whatever comes to my mind”* (Mrs. J, para. 132). She relaxed through *“Yoga, already ten years. So that my agility and so still remains. So this is an hour which is then belonging to me”* (Mrs. J, para. 148). *“My resting point is actually the cat. […] She purrs with patience and you really drive down. Silence. Calmness. Above all she listens and does not contradict”* (Mrs. J, para. 45–47).

Mrs. J looked to the future with worry. *“Just with increasing age you fear it will become worse. Therefore you must always say, no progress must already be considered as progress”* (Mrs. J, para. 126).

Mrs. J felt emotionally and temporally burdened. She did not engage professional care and assistance services. By searching for ‘concentrated relaxation’, ‘emotional release’, ‘attention and care’ and as well as ‘social withdrawal’, she applied the coping dimension ‘seeking attention and care’. However, her statements revealed her main coping dimension to be ‘negative emotional’ (i.e., ‘rumination’, ‘self-pity’, ‘resignation’ and ‘release of anger’). There was no recognisable ‘diverting’ coping style. It appears difficult for Mrs. J to integrate the current situation into her life.

## Discussion

This study investigated the relationship between caregiver burden experience and the coping strategies of spouses of geriatric patients by constructing a typology using a mixed method design. Results indicate that coping behaviour changes with an increasing perception of burden and stress. As shown in Table 1, in most cases our participants had a mix of coping strategies of different dimensions, but one seems to be the most important and was then grouped into the type.

Participants fitting Type A, “The Caring Partner”, focused on the relationship with the partner. Decision-making was done together and the “We” was in focus. Additionally, the needs and the well-being of the partner to be cared for were the center of attention. Kaplan has described a similar type as “Til Death Do Us Parts”, in a typology of partners of people with Alzheimer’s disease in a care institution [28]. The “We” aspect has also been described in the so-called “Adapter” type [27], although here adult children also formed part of the “We” regarding social support.

Spouses who described their caregiving burden as being little to moderate tended to apply stress-reducing coping styles. Gunzelmann et al. found that emotional support is a predictor of coping by ‘diverting’ [24]. This is consistent with our findings, although we could not find a connection between formal/informal support and the caregiver burden or stress. With Type A, even the care-receiving spouses were considered a socio-emotional support and more benefits of caregiving were identified. The EE score was also rather low.

Those fitting Type B, “The Affected Manager”, felt more burdened and stressed. The burden was attributed less to the care recipient than to the changed living situation. The positive view of the relationship was based on the shared past. The well-being of the partner was at the center of activities. It bothered the caregiver when the situation could not be managed alone and outside support was difficult to accept. This difficulty was also reflected in an increased EOI score. Caregivers fitting this type tried to increase their self-confidence especially through coping by ‘seeking attention and care’. Similarities can be found in the “Case manager” type by Davis et al., which described spouses who managed the situation as a task to be done and organised it primarily on their own, but without involving their partner [27].

Among all three types, participants fitting Type C, “The Desperate Overburdened”, reported feeling the greatest sense of burden and stress. Causes given were both the changed living situation and the changed partner. The life of the caregiver receded into the background, social contacts were rare, and the feeling of having to decide everything alone dominated. The “We” rarely appeared in the statements, but the “I” dominated. The aspect of “being alone” can also be found in the “Struggler” [27] and “Struggling” type [29], which incidentally also reported the highest stress levels. Unlike our sample, “being alone” for “Strugglers” was caused by resistance to caring for a partner with dementia. In the present study, a perceived lack of cooperation and missing emotional support and recognition were mentioned as causes. The relationship to the partner was considered to be rather distanced. The ‘negative emotional’ dimension of coping was predominant, which could also be due to a perceived low level of control [23, 24].

For all participants in the present study, social contacts were reduced and essentially limited to the immediate family circle or, where appropriate, contacts with the (church-) community. This social withdrawal, however, was interpreted differently depending on type. With Type A, withdrawal tended to take place in the context of focusing on one’s own partner and the time spent together was experienced as very valuable. With Type C, however, withdrawal was more to focus on oneself, trying to protect oneself from being overburdened.

Li observed that the kind of family caregiver worry during the hospital stay of an older relative is related not only to the patient’s conditions but also to the ability of the family to provide post-hospital care [40]. This can also be found in the presented typology. In Type A, the main worry was about the sick partner. With Type C, the stress of the organisational workload associated with the hospital stay and the worry about providing care after discharge were paramount. Both aspects could be found in Type B.

The present study also tended to confirm that a higher care burden is associated with poorer physical and emotional health [6, 16]. Hessel et al. [23] associated a more positive evaluation of one’s health with the coping dimension ‘diverting’ and poorer health with the coping dimension ‘seeking attention and care’. This condition was also reflected in our typology. The findings on differences between men and women are heterogeneous [11, 15]. We could not find any gender differences between in construction of types. It has been observed, however, that caregiving motivation influences caregiver burden [41]. In this study, obligation motives were associated with a higher caregiver burden and affection motives were associated with a lower burden.

Daley et al. examined caregiving spouses of patients with Alzheimer’s disease [26]. Utilizing Kaplan’s couplehood typology [28], caregiving spouses were divided into two groups based on expressed closeness to the partner: “We/Us” and “I/Me”. They found no significant differences between the two groups in terms of cognition and functionality of the care recipient, or regarding levels of anxiety, depression, burden or satisfaction with the relationship. The authors, however, noted that relationship satisfaction was higher in the “We/Us” group. Monin et al. identified a significant correlation between relationship satisfaction, depressive symptoms, and self-reported health status [42]. Daley et al. also showed that in the “We/Us” group, positive aspects were more often mentioned regarding the care situation [26]. This was also observed in the current study. In regular increments, the positive aspects of care were less frequently mentioned moving from Type A to Type C. Furthermore, no association between type and attributes of the care recipient could be found. Based on the interviews, “We/Us” was more dominant in Type A and the “I/Me” was more dominant in Type C. Both aspects were expressed by Type B, in which the “I/Me” managed the “We/Us” of the couple’s relationship.

There are some limitations to be mentioned. The data are limited due to the small sample size. However, interpretive consistency could be achieved using analytical generalizations/case-to-case transfers despite a small sample size [43]. Nine spouses of geriatric patients in a single hospital were interviewed. Therefore, no attempt at a definitive typology of caregiving spouses can be made. For example, there were no couples living with another relative in the same household or in the immediate neighbourhood in the current sample. Such a situation would certainly have influenced the typology. With regard to characteristics such as level of care, gender, duration of care, degree of care or type support, no correlations were found in this typology. Larger samples in further studies are necessary for sophisticating this typology. The equal distribution in the study further precludes the formation of a conclusion regarding the actual distribution of types. Moreover, although no general correlation was found based on the sample, the interviews took place at different times in relation to the last hospital stay. This might have influenced the experience of burden and stress.

The analysis of qualitative data is open to various interpretations. An attempt was made to limit this weakness through a structured approach and a high level of transparency to data collection, analysis and interpretation. The mixed method design thus helped in the search for a deeper understanding of social reality in the context of geriatric care.

## Conclusions

The development of this new typology of caregiving spouses could help health care professionals better understand caregiving arrangements. Previous studies have focused on spouses with partners with neurodegenerative diseases, particularly dementia. The current study revealed that partners of patients without dementia can also be exposed to higher stress levels. More relevant than an underlying disease or the degree of dependency on long-term care seems to be the quality of the relationship to the care recipient. In this respect, members of the health care professions should pay attention to how the caregiving partners talk about their situation: Are they more likely to speak of “we/us”, or “I/me”, or are both forms present?

The transactional coping model of Lazarus and Folkmann has been described as a dynamic process [19]. Dealing with stress and strain can be influenced by a reappraisal of the situation and the coping strategies. Thus, caregivers of Type C could be supported in identifying positive aspects of care and the resources of their partner. They could be encouraged to establish free space for themselves and to use this space in a more valuable way to strengthen their self-efficacy.

Type B spouses could be encouraged to strengthen self-efficacy as well as use and expand existing coping strategies from the dimensions ‘diverting’ and ‘seeking attention and care’. As there is usually a positive relationship with the partner and common interests, this should be recognised and used as a resource. At the same time, caregivers should realise their own limits without feeling that they have failed. Type B should be empowered to accept support, especially in the organisation and planning of care.

In the case of Type A spouses, it seems particularly important to view the two spouses as a unit and accordingly involve both partners in counselling and care. These caring spouses can also have a caregiver burden and here it is important to ensure that do not overburden themselves and accept support in good time.

## Supplementary Information

**Additional file 1.** Interview guide.

## Data Availability

The datasets generated and analysed during the current study are not publicly available due to participant confidentiality but are available from the corresponding author on reasonable request.

## Abbreviations

BEFO: Bernese Coping Modes
EE: Expressed emotions
EOI: Emotional over-involvement
CC: Criticism
PSQ: Perceived Stress Questionnaire
TIGER: Transsectoral Intervention Program for Improvement of Geriatric Care in Regensburg Study
ZBI: Zarit Burden Interview
