# Supplementary material for: A typology of caregiving spouses of geriatric patients without dementia: caring, worried, desperate
Source: BMC Geriatr. 2021 Sep 6;21:483. doi: 10.1186/s12877-021-02425-1 (PMC8419985; doi:10.1186/s12877-021-02425-1)
Supplement: Supplementary file 1 — Additional file 1. Interview guide. [file 12877_2021_2425_MOESM1_ESM.pdf]

A Typology of caregiving spouses of geriatric patients without dementia: Caring, Worried, Desperate

Interview guide

Suggested questions/interview topics	Additional Prompts
**Introduction**<br>• Acknowledgement for time and participation<br>• Information about aim of the research project<br>• Information and consent to the recording<br>• Assurance of anonymity	
**Interview**<br>• I`d like you to tell me about yourself. Tell me about the most important stages in your life.<br>• I am particularly interested in caregiving spouses. Can you tell me about a typical day with your wife/husband, for example about yesterday? Start with getting up in the morning.<br>• How did it come about that you have taken over the care of your partner?<br>• How would it be if someone else took over the care? Would a nursing home be an alternative?<br>• The Media often talks about the burden of caring for relatives - how is it for you?"<br>• I have also read about positive experiences and aspects of taking over care. What are your experiences in this regard?<br>• You told me about your daily routine and what you do for your wife/husband. Many caregivers in your situation would like support - so how about you?<br>• People who have demanding or stressful experiences often have a very individual approach to dealing with them. They have developed their own strategies - how about you?<br>• What are your thoughts about you and your wife's/husband's future?"	Can you elaborate on this...<br>How do you feel...<br>Can you give me an example ...<br>Can you describe it to me in detail?<br>What happened…<br>What did you think/feel…<br>What does it mean to you…<br>What triggers this in you?
**Debriefing**<br>• Is there anything else you would like to share with me regarding your situation?<br>• Closing words	
